# Redescriptions, Lectotype Designations, New Synonyms and New Geographic Records for the “Tiger” Species of *Mycotretus* Lacordaire, 1842 (Coleoptera: Erotylidae: Tritomini)

**DOI:** 10.3390/insects9040168

**Published:** 2018-11-22

**Authors:** Italo S. C. Pecci-Maddalena, Cristiano Lopes-Andrade

**Affiliations:** 1 Programa de Pós-Graduação em Ecologia, Departamento de Biologia Geral, Universidade Federal de Viçosa, Viçosa, Minas Gerais 36570-900, Brazil; 2 Laboratório de Sistemática e Biologia de Coleoptera, Departamento de Biologia Animal, Universidade Federal de Viçosa, Viçosa, Minas Gerais 36570-900, Brazil; ciidae@gmail.com

**Keywords:** pleasing fungus beetles, Neotropical region, taxonomy, intraspecific variation, morphology

## Abstract

The Neotropical *Mycotretus* Lacordaire, 1842 is one of the largest and most widespread genera of the Erotylidae, encompassing more than 200 described species. Among the species with a similar body coloration, there is a “group” of six valid species—called here the “tiger” *Mycotretus*—that possess several pronotal and elytral black spots, as follows: *M. tigrinus* (Olivier, 1792); *M. multimaculatus* Taschenberg, 1870; *M. centralis* Arrow, 1909; *M. tigrinoides* Mader, 1942; *M. tigripennis* Mader, 1942; and *M. prioteloides* Mader, 1942. Different from any other *Mycotretus* with spots, the spots of the “tiger” *Mycotretus* are numerous and are not bilaterally symmetrical in pattern. Here, new geographical records, diagnoses and redescriptions are provided for *M. tigrinus*, *M. centralis*, *M. tigrinoides*, *M. tigripennis* and *M. prioteloides*, including the first descriptions of their male and female terminalia. Lectotypes are designated for *M. multimaculatus*, *M. centralis*, and *M.* *leopardus*. *Mycotretus multimaculatus* and *M. tigrinus pardalis* Crotch, 1876 are proposed as new junior synonyms of *M. tigrinus*. Additionally, the authorship of the name *M. leopardus* is attributed to Crotch, 1876, because he was the first author to provide a description for that taxon, and the synonymy of *M. leopardus* and *M. conspersus* (Germar, 1824) with *M. tigrinus* (Olivier, 1792) is confirmed.

## 1. Introduction

The Neotropical *Mycotretus* Lacordaire, 1842 is one of the largest genera in the family Erotylidae, encompassing more than 200 described species widespread in the Neotropical region [1,2,3]. The genus is taxonomically problematic as most species are known only from older descriptions [1,4,5], information about male and female terminalia is scarce [3,6,7], and no taxonomic revision has been provided to date. Data on host fungi of *Mycotretus* are scarce and records are too few to discuss host fungus specialization. Most other Erotylinae species of *Mycotretus* feed on basidiomycete fungi, with host records in the families Pleurotaceae, Polyporaceae and Mycenaceae [3]. There are records of two different species of *Mycotretus* co-occurring in the same host fungus, for instance in the case of *M. chilensis* Crotch, 1876 and *M. trifasciatus* Guérin, 1956 co-occurring in the fungi *Mycena* sp. (Mycenaceae) and *Lentinus brumalis* (Polyporaceae) [3].

Dorsal color patterns are extremely variable in *Mycotretus*, ranging from completely monochromic to species with several spots, bands or stripes, especially on the pronotum and elytra [1,3]. Among *Mycotretus* there is an assemblage of six valid species—called the “tiger” *Mycotretus* from now on in the text—recognized by the presence of many pronotal and elytral black spots, usually sparsely distributed in no discernable pattern, as follows: *M. tigrinus* (Olivier, 1792); *M. multimaculatus* Taschenberg, 1870; *M. centralis* Arrow, 1909; *M. tigrinoides* Mader, 1942; *M. tigripennis* Mader, 1942; and *M. prioteloides* Mader, 1942. The first one, *M. tigrinus*, was described from Suriname and is common and widespread in the Neotropical region [2]. Lacordaire [4] synonymized *M. conspersus* (Germar, 1824) with *M. tigrinus*, and Crotch [5], described *M. tigrinus pardalis* Crotch, 1876, originally as a new “variety”. Later, Kuhnt [8] proposed *M. leopardus* Gorham, 1888 as a synonym of *M. tigrinus* (see Alvarenga [2]).

Two other “tiger” *Mycotretus*, *M. multimaculatus* from Colombia and *M. centralis* from Central America, resemble *M. tigrinus* in coloration and body shape. *Mycotretus centralis* was described based on individuals first identified by Gorham [1] as simple variations of *M. tigrinus*. It is worth noting that Arrow [9] considered such specimens as distinct from *M. tigrinus*, but did not provide a satisfactory description, mentioning only that “Besides the differences noted by Mr. Gorham, it is a rather more massive species and the metasternum, which is well punctured in *M. tigrinus* is very smooth”. Therefore, the limits between *M. centralis* and *M. tigrinus* remained unclear to date. The other three *Mycotretus* species treated here were described by Mader [10]: *M. tigrinoides* from Ocobamba (Peru), *M. tigripennis* from Santa Inéz (Ecuador) and *M. prioteloides* from Coroico (Bolivia) and Calango (Peru). The former two descriptions (*M. tigrinoides*, *M. tigripennis*) were based only on their respective holotype, while the latter one (*M. prioteloides*) was based on two individuals.

Here, five species are redescribed, *M. tigrinus*, *M. centralis*, *M. tigrinoides*, *M. tigripennis* and *M. prioteloides*, including the first descriptions of their male and female terminalia, and new geographical records are presented. Lectotypes are designated for *M. multimaculatus*, *M. centralis* and *M. leopardus*. *Mycotretus multimaculatus* and *M. tigrinus pardalis* are proposed as junior synonyms of *M. tigrinus*. We also discuss minor taxonomic results and the morphological affinities of the species treated here with other species in the genus. Additionally, the authorship of the name *Mycotretus leopardus* is attributed to Crotch [5] and the synonymies of *M. leopardus* and *M. conspersus* with *M. tigrinus* are confirmed.

## 2. Material and Methods 

Dissection, photography and measurement of specimens followed the methods provided by Pecci-Maddalena and Lopes-Andrade [3]. Transcription of labels followed Pecci-Maddalena and Lopes-Andrade [11]. The distribution map was created using latitude and longitude coordinates estimated by tracking localities in the online database GeoNames [12] and plotted on a map in the freeware Q Geographic Information System (QGIS 2.12.2). Localities in the maps were represented by an Arabic numeral using an image editing program. 

**Figure 1:**
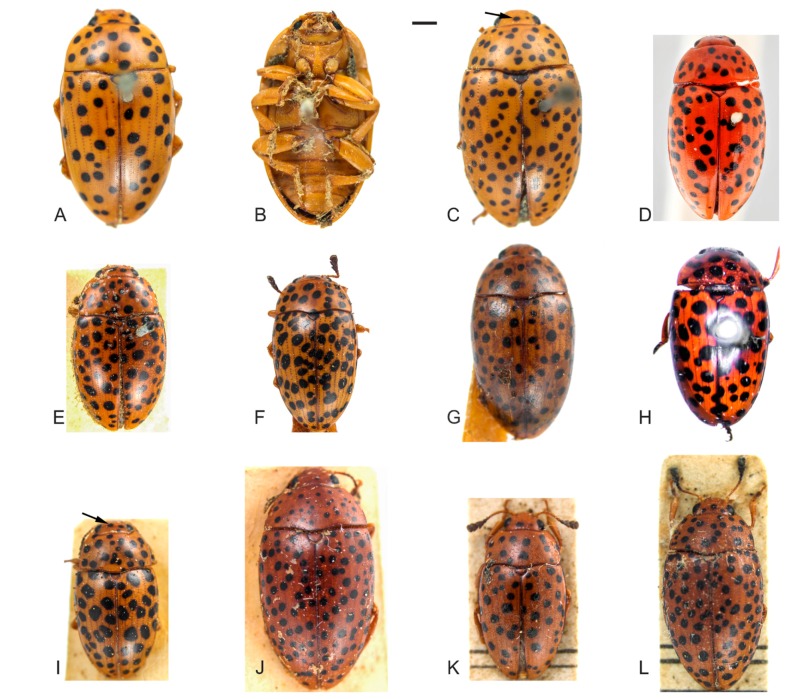
(**A**–**L**) Specimens of *Mycotretus tigrinus* Olivier, 1792 from different localities: (**A**–**B**) male, dorsal and ventral view, respectively (Corupá, Santa Catarina (SC)). (**C**–**L**) dorsal view: (**C**) female, arrow showing a spot on the head (Corupá, SC); (**D**) a “topotype” deposited in the Royal Belgian Institute of Natural Sciences (RBINS) (Suriname); (**E**) male (Tefé, Amazonas (AM), Brazil); (**F**) male (Linhares, Espírito Santo (ES), Brazil); (**G**) female (Parque Nacional do Xingu, Mato Grosso (MT), Brazil); (**H**) lectotype of *M. multimaculatus* Taschenberg, 1870 (Colombia); (**I**) male, arrow showing spots on the head (Buena Vista, Bolivia); (**J**) female (Buena Vista, Bolivia); (**K**) male (Chapare, Bolivia); (**L**) female (Chapare, Bolivia). Scale bars: **A**–**G**, **I**–**L** = 1 mm; H, see Section 2.

Terms for external morphology follow Lawrence et al. [13] and McHugh et al. [14], and those for color pattern follow Skelley [15]. Descriptions of mouthparts and abdominal terminalia were based on Węgrzynowicz [16]. The term “flagellum” refers to a male genitalia structure with two interconnected elements: “head” and “virga” [16]. The following abbreviations are used: TL, total length (= elytral length + pronotal length along midline; head not included); EW, greatest elytral width. The ratio TL/EW indicates the degree of body elongation. The heading of each species redescribed below includes only a bibliographic citation of the original description and type locality, as far as new combinations and synonyms when applicable. Complete bibliographic citations are available in Alvarenga [2] and are not repeated here. A scale bar was not included in Figure 1H (lectotype of *M. multimaculatus*, here designated) because the number of examined specimens used to determine species length (6 mm) mentioned in the original description [17] is unknown. Due to the great length variation in *M. tigrinus*, and in order to make it clear for readers, the other specimens shown in Figure 1 are all in the same scale.

The following federal states of Brazil (official abbreviations in parentheses) are cited in the text: Amazonas (AM); Distrito Federal (DF); Espírito Santo (ES); Góias (GO); Mato Grosso (MT); Minas Gerais (MG); Pará (PA); Paraná (PR); Rio de Janeiro (RJ); Rio Grande do Sul (RS); Santa Catarina (SC); São Paulo (SP). The denomination “Reprêsa Rio Grande” (present on the labels of several specimens studied here) was given to only one of several streams dammed at the time. Currently, it is part of the set of reservoirs of the “Ribeirão das Lajes” dam and its whole area is an ecological reserve. The reservoir comprises areas in Piraí (RJ) and Rio Claro (RJ). Moacyr Alvarenga collected beetles in the former (personal communication by Ayr de Moura Bello, 2018).

The specimens studied here belong to the following collections:

BMNH The Natural History Museum (London, UK)
CAMB Coleção Ayr de Moura Bello (Rio de Janeiro, RJ, Brazil)
CELC Coleção Entomológica do Laboratório de Sistemática e Biologia de Coleoptera (Viçosa, MG, Brazil)
DZUP Coleção Entomológica Padre Jesus Santiago Moure, Universidade Federal do Paraná (Curitiba, PR, Brazil)
MCNZ Fundação Zoobotânica do Rio Grande do Sul (Porto Alegre, RS, Brazil)
MNHN Muséum National d’Histoire Naturelle (Paris, France)
MNRJ Museu Nacional (Universidade Federal do Rio de Janeiro, Rio de Janeiro, RJ, Brazil)
MRSN Museo Regionale di Scienze Naturali (Torino, Italy)
MZSP Museu de Zoologia, Universidade de São Paulo (São Paulo, SP, Brazil)
NMBS Naturhistorisches Museum Basel (Basel, Switzerland)
RBINS Royal Belgian Institute of Natural Sciences (Brussels, Belgium)
SDEI Senckenberg Deutsches Entomologisches Institut (Müncheberg, Germany)
UMZC University Museum of Zoology Cambridge (Camdridge, UK)
ZNS Zentralmagazin Naturwissenschaftlicher Sammlungen (Halle, Germany)

## 3. Results

For nomenclatural stability, the lectotype of *M. centralis*, *M. leopardus* and *M. multimaculatus* are designated below. Based on original descriptions, historical material examined, external morphology and coloration pattern of adults, we: (i) propose *M. multimaculatus* and *M. tigrinus pardalis* as junior synonyms of *M. tigrinus*; (ii) attribute the authorship of the name *M. leopardus* to Crotch [5], because he was the first author to provide a description for that taxon; and (iii) confirm the synonymy of *M. leopardus* and *M. conspersus* with *M. tigrinus*. The redescriptions of *M. tigrinus*, *M. centralis*, *M. tigrinoides*, *M. tigripennis* and *M. prioteloides* are provided below in chronological order.

### 3.1. Mycotretus tigrinus (Olivier, 1792)

**Figure 2:**
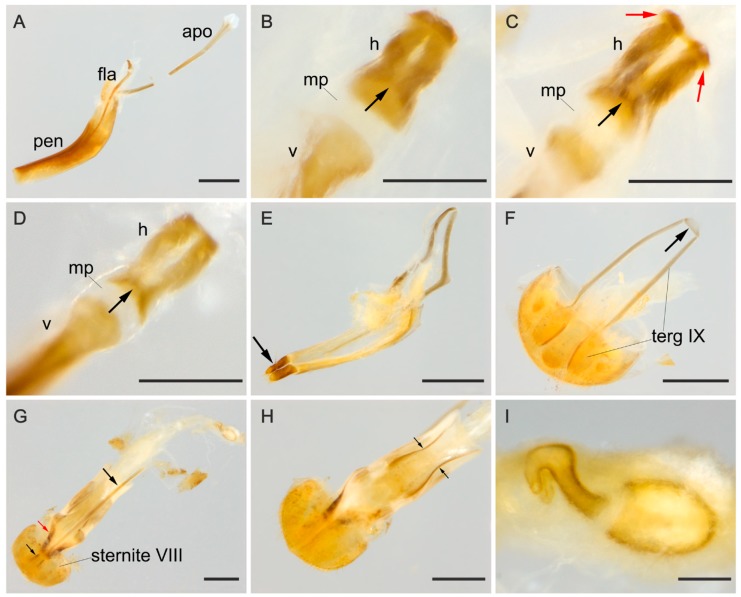
(**A**–**F**) Male abdominal terminalia of *Mycotretus tigrinus* Oliver, 1792 from different localities: (**A**–**B,E**–**F**) Tefé, AM, Brazil; (**C**) Linhares, ES, Brazil; (**D**) Rio Toro, Peru. (**A**) lateral view of the aedeagus, showing apophyses (apo), flagellum (fla) and penis (pen). (**B**–**D**) Dorsal view of flagellum showing: head (h), anterior tip of virga (v) and the membranous portion (mp); black arrows showing a posterior arcuate sclerotization and red arrows (in C) showing an anterior outer sclerotization, forming two narrowed tips. (**E**) Tegmen, arrow showing parameres; (**F**) abdominal segments VIII–X: laterotergite IX (terg IX), arrow showing the sclerite at the anteroventral edge of segment IX. (**G**–**I**) Female abdominal terminalia of a specimen from Parque Nacional do Xingu, MT, Brazil: (**G**) dorsal view, with small black and red arrows showing a gonostylus and a gonocoxite, respectively; big black arrow showing the median strut of sternite VIII; (**H**) ventral view, arrows showing baculi of paraprocts; (**I**) spermatheca. Scale bars: **A**, **E**–**H** = 0.5 mm; **B**–**D**, **I** = 0.1 mm.

**Figure 3:**
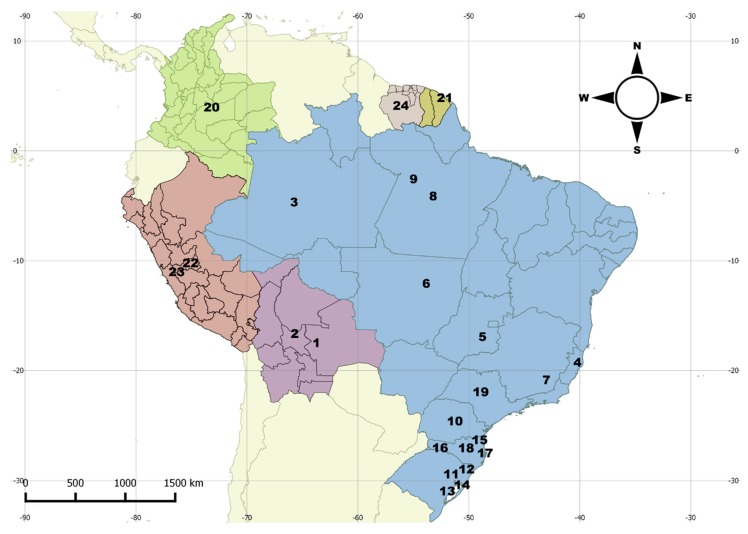
Distribution map for *Mycotretus tigrinus* (Olivier, 1792). Bolivia (1–2): 1—Buena Vista, 2—Chapare. Brazil (3–19): 3—Tefé (AM); 4—Linhares (ES); 5—Vianopólis (GO); 6—Parque Nacional do Xingu (MT); 7—Viçosa (MG); 8—State of Pará (undetermined locality), 9—Santarém (PA); 10—Reserva (PR); 11—Novo Hamburgo, 12—São Francisco de Paula, 13—Tapes, 14—Viamão (RS); 15—Corupá, 16—Nova Teutônia, 17—São Pedro de Alcântara, Santa Catarina (undetermined locality) (SC); 19—São Paulo (SP). Colombia: 20 (undetermined locality). French Guiana: 21—Cayena. Peru: 22 (undetermined locality), 23—Río Toro. Suriname: 24 (undetermined locality).

Figure 1, Figure 2 and Figure 3.

*Erotylus tigrinus* Olivier 1792: 437 [18]. Type locality: Suriname. 

*Mycotretus tigrinus* (Olivier, 1792). Lacordaire 1842: 145 [4] (new combination).

*Mycotretus leopardus* Crotch 1876 [5]. Type locality: Peru. Crotch 1876: 451 (junior synonym).

*Mycotretus multimaculatus* Taschenberg 1870: 197 [17]. Type locality: Colombia, **new synonym**. 

*Mycotretus tigrinus pardalis* Crotch 1876: 451 [5] (as a variety). Type locality: Ecuador, **new synonym**.

**Diagnosis.** Dorsal coloration with several circular and subcircular black spots, extremely variable in size and number and asymmetrically distributed. Penile flagellum slightly elongated (approximately 0.7 × the length of penis), slightly sinuous and with a membranous portion between its virga and head. Head of flagellum sclerotized and elongated, with an arcuate sclerotization posteriorly and an inflection at the basal half of the lateral edges. Inner contours slightly separated; anterior edge with outer sclerotization, more or less prominent and, sometimes, forming two outer, narrowed and lateral tips.

**Redescription**. Length (in mm) = 4.71–8.26 (6.87 ± 0.94, *n* = 23). Body elongate, slightly oval, widest at the anterior third of elytra, TL/EW = 1.57–1.80 (1.70 ± 0.05), glabrous and glossy, dorsal and ventral coloration homogeneously yellowish or reddish-brown (Figure 1A–L). Mouthparts with same background color as body, mandibles apex blackish and with two teeth; mentum plate pentagonal, with strongly sclerotized margin; antennae yellowish or reddish-brown, last antennomeres blackish. Scutellar shield yellowish, reddish-brown or blackish, glabrous and bearing few punctures. Dorsal coloration: head lacking or, usually, with one to four asymmetrical subcircular black spots (Figure 1C,I, arrow); pronotum with several circular and subcircular black spots (Figure 1A,C–L), extremely variable in size and number (usually more than ten), and asymmetrically distributed in all examined specimens (except for the specimen from Río Toro, Peru with more symmetrical pronotal spots). Elytral coloration similar to that of pronotum, with several circular and, usually, free and sparsely distributed spots (Figure 1A,C–L). 

**Male terminalia.** (Figure 2A–F). Penis (Figure 2A, pen) slightly elongate and curved; basal portion with a short sclerotized projection linked to apophyses; internal sac with well-developed and slightly elongated flagellum (Figure 2A, fla), 0.7 × the length of penis (*n* = 3), with slight sinuosity and a membranous portion between the virga and the head of the flagellum (Figure 2B–D, mp), head of flagellum (Figure 2B–D) sclerotized and elongated, with arcuate sclerotization posteriorly (Figure 2B–D, black arrow) and inflection at the basal half of the lateral edges. Inner contours slightly separated; anterior edge with outer sclerotization, more or less prominent and, in some individuals, forming two outer, narrowed and lateral tips (Figure 2C, red arrows). Apophyses (Figure 2A, apo) 1.12 × as long as penis (*n* = 2). Tegmen sclerotized (Figure 2E); parameres reduced and sclerotized, with densely pubescent outgrowths, slightly dilated, narrowed and acute at apex (Figure 2E, arrow). Tergite VIII, sclerotized with sparsely distributed bristles and sternite VIII slightly sclerotized. Laterotergite IX sclerotized, posteriorly elongated and pubescent, outer contours angulated (Figure 2F, terg IX); anteroventral edge with paired and subparallel lateral struts, connected at its anterior tip by small transverse, slightly sclerotized sclerite (Figure 2F, arrow). Posterior edge of sternite IX, sclerotized, undivided, outer contour rounded; anteriorly membranous. Tergite X, sclerotized, anterior edge with sparsely distributed bristles. 

**Female terminalia.** (Figure 2G–I). Gonostyli and gonocoxites strongly sclerotized (Figure 2G, black and red arrows, respectively, under sternite VIII), baculi of paraprocts sclerotized and sinuous (Figure 2H, arrows); spermatheca oval and sclerotized (Figure 2I). Tergite VIII sclerotized and sternite VIII with a conspicuous median strut (Figure 2G, sternite VIII, big black arrow).

**Distribution.** Northern to southern Neotropical region (Figure 3).

**Remarks**. (1) As occurs for other Olivier primary types [15,19], the repository of the type of *M. tigrinus* is unknown. We identified this species based on the original description, early redescriptions [1,20], a series of specimens from several museums, and images of two “topotypes” from the RBINS (Figure 1D, dorsal view of one specimen). (2) Alvarenga [2] stated that the repository of the type of *M. multimaculatus* was unknown. However, according to Horn et al. [21], Taschenberg specimens would be housed in the ZNS. The specimens are indeed in the ZNS and images were sent to us by the curator (Figure 1H, lectotype). The Taschenberg specimen clearly shows the same color pattern of the other *M. tigrinus* examined by us and we synonymize it with *M. tigrinus.* (3) Another examined specimen from Peru, the “type of *M. leopardus*”, is kept in the UMZC. The authorship of *M. leopardus* had been attributed to Gorham [1] by previous authors [2,8,22], although Gorham attributed it to Kirsch (see Gorham [1]). However, we verified that the first author to provide a diagnosis for *M. leopardus* (and “in litteris” by Kirsh) was Crotch [5], who also published the new name as a synonym of *M. tigrinus*. In this case, according to the International Code of Zoological Nomenclature (ICZN, Article 50.7, p. 53 [23]), the authorship of names first published as junior synonyms is attributed to “the person who published it as a synonym, even if some other originator is cited, and is not the person who subsequently adopted it as a valid name”. It is no coincidence that the type specimen of *M. leopardus* is in UMZC, where the remaining Crotch types are kept. Therefore, here we attribute the name *M. leopardus* to Crotch [5] and confirm the synonymy of *M. leopardus* with *M. tigrinus* proposed by him. (4) We have not located the type specimen of *M. tigrinus pardalis* in the UMZC, or in other European museums visited or consulted by us, and there is a great chance that the type is lost. Based on the original description and historical material examined, we conclude that *M. tigrinus pardalis,* originally described as a variety, is merely an intraspecific variation of *M. tigrinus*. (5) The type of *M. conspersus* was also not located, but based on the historical material examined, especially on an old specimen from MNHN identified as *M. conspersus* (see Material examined below), aside from the comments provided by Lacordaire [4], we confirm the synonymy of *M. conspersus* with *M. tigrinus.*

**Material examined**.** Lectotype of *M. multimaculatus *Taschenberg, 1870, here designated** (ZNS) “multimaculatus, Zeitschr. 1870. Colomb. Wallis [green label, handwritten, box label] \ LECTOTYPE *Mycotretus multimaculatus *Taschenberg, 1870 det. I. Pecci-Maddalena 2017 [red label, printed]”; 1 specimen (ZNS) “multimaculatus, Zeitschr. 1870. Colomb. Wallis [green label, handwritten, box label]”; 1 specimen (UMZC) “Cayen [green label, handwritten] \ TYPE [printed, crossed out] tigrinus, coll Reiche [handwritten]”; 1 specimen (UMZC) “So Pau [handwritten, São Paulo?]”; 2 specimens (UMZC) “Chevr. [printed]”; 2 specimens (UMZC) “green label \ Bates [printed]”; **lectotype of *M. leopardus *Crotch, 1876, here designated **(UMZC) “TYPE [blue label, printed] \ TYPE [printed] leopardus Peru [handwritten]”; 1 male (BMNH, dissected) “Ega [handwritten], 57, 125 [handwritten on label back] \ *M. tigrinus* Oliv. [handwritten]”; 1 specimen (BMNH) “Santarem [front] 53, 92 [back] [handwritten, disc-shaped label]”; 1 specimen (BMNH) “Demarara [handwritten, Demerara?] \ 1419 [printed] \ *tigrinus Ol.*, *1419*. *S Am *[handwritten]”; 1 specimen (BMNH) “*W Burnett*, *Brasil *[? handwritten] \ Ent. Club. 44-12. [printed]”; 1 specimen (BMNH) “Rio Grande, 84 a 8 [handwritten]”; 1 specimen (BMNH) “Rio Grande, 86-9. [handwritten]” 1 specimen (BMNH) “Cayenne [front] 58, 74 [back] [handwritten]”; 1 specimen (BMNH) “Para [handwritten, disc-shaped label] \ Mycotretus tigrinus Ol [handwritten] \ Pascoe Coll. 93–60 [printed]”; 1 specimen (BMNH) “Buenavista, BOLIVIA, II-IV, 1925 [printed] \ Provincia, d’SARA, 1700 ft. [printed] \ ex coll. F. Mason. B.M.1926–296. [printed]”; 1 specimen (MRSN) “latreille [handwritten]”; 1 specimen (MRSN) “Lacordaire [handwritten]”; 1 specimen (MRSN) “Brasilia Truqui [printed]”; 1 specimen (SDEI) “Chaucham [? Chauchamayo? handwritten] \ Schenkling det. [printed]”; 1 specimen (RBINS) “Mycotretus tigrinus, Surinam Oliv [handwritten] \ Coll. R. I. Sc. N. B., Surinam, Coll. Chapuis [printed]”; 1 specimen (RBINS) “det …., MYCOTRETUS [printed] *tigrinus Ol*. [handwritten] \ Coll. R. I. Sc. N. B., Surinam, Coll. Chapuis [printed]”; 1 specimen (MNHN) “4125, 33T [? disc-shaped label]”; 1 specimen (MNHN) “prov. De S^ta^ Catherin, bords dela mer, 1820 [handwritten, disc-shaped label] \ *Micotretus conspersus Ger *[handwritten] \ tigrinus [handwritten]”; 1 specimen (MNHN) “7151, 34. [?, handwritten, disc-shaped label] \ 2192 [?, handwritten]”; 1 specimen (MNHN) “6546, 34. [handwritten, disc-shaped label] \ 1947 [?, handwritten]”; 1 specimen (MNHN) “tigrinus, Cayenne [handwritten]”; 1 specimen (MZSP) “III. 1930 [handwritten], Goyaz [printed], Viannopolis, Coll R Stitr [handwritten] \ *Mycotretus tigrinus Ol. *[handwritten]”; 1 male (MNRJ, dissected) “Coleção M. Alvarenga [printed] \ Peru Rio Toro [printed] \ *Mycotretus multimaculatus Tasch., 1870 *[handwritten] M. Alvarenga det. 1971 [printed]”; 1 female (DZUP, dissected) “Coleção M. Alvarenga [printed] \ *Buenavista, Bolivia, iii/viii. 1950 *[handwritten] \ DZUP 371251 [printed]”; 1 male (DZUP, dissected) “Coleção M. Alvarenga [printed] \ *Buenavista, Bolivia, iii/viii. 1950 *[handwritten] \ DZUP 371252 [printed]”; 1 male (DZUP, dissected) “Coleção M. Alvarenga [printed] \ Chapare 400 m, Bolivia [printed], *viii. 1954 *[handwritten], R. Zischka [printed] \ DZUP 371231”; 1 female (DZUP, dissected) “Coleção M. Alvarenga [printed] \ Chapare 400 m, Bolivia [printed], *viii. 1954 *[handwritten], R. Zischka [printed] \ DZUP 371232”; 1 male (DZUP, dissected) “Coleção M. Alvarenga [printed] \ Chapare 400 m, Bolivia [printed], *viii. 1954 *[handwritten], R. Zischka [printed] \ DZUP 371237”; 1 female (DZUP, dissected) “Coleção M. Alvarenga [printed] \ Jacaré P.N. Xingu, M. Grosso Brasil, XI-1961, Alvarenga e Werner [printed] \ DZUP 371247 [printed]”; 1 male (DZUP, dissected) “Coleção M. Alvarenga [printed] \ Parque Sooretama, LINHARES E. Santo, Brasil X-1962, M. Alvarenga leg. [printed] \ DZUP 371259 [printed]”; 1 female (DZUP, dissected) “Coleção M. Alvarenga [printed] \ Buenavista, Bolivia, 1956, A. Martinez [handwritten] \ DZUP 371254 [printed]”; 1 female (DZUP, dissected) “Coleção M. Alvarenga [printed] \ Jacaré P.N. Xingu, M. Grosso Brasil, XI-1961, Alvarenga e Werner [printed] \ DZUP 371246 [printed]”; 1 male (DZUP, dissected) “Coleção M. Alvarenga [printed] \ Parque Sooretama, LINHARES E. Santo, Brasil X-1962, M. Alvarenga leg. [printed] \ DZUP 371258 [printed]”; 1 male (DZUP, dissected) “Coleção M. Alvarenga [printed] \ COLEÇÃO CAMPOS SEABRA [printed] \ *Mycotretus tigrinus Ol*. [handwritten] J. Guerin det. 19[printed]53 [handwritten] \ CORUPA, Santa Catarina BRASIL [printed] X-1951 [handwritten] ANTON MALLER [printed] \ DZUP 371286 [printed]”; 1 female (DZUP, dissected) “Coleção M. Alvarenga [printed] \ COLEÇÃO CAMPOS SEABRA [printed] \ *Mycotretus tigrinus Ol*. [handwritten] J. Guerin det. 19[printed]53 [handwritten] \ CORUPA, S. Catarina BRASIL [printed] I [handwritten] A. MALLER [printed] \ DZUP 371285 [printed]”; 1 female (DZUP, dissected) “II. [handwritten] 196[printed]5[handwritten], Brasilien, Nova Teutonia, 27°11′B, 52°23′L, Fritz Plaumann, 300.500 m [printed] \ DZUP 125458 [printed]”; 1 female (CELC) “Viçosa/MG/Brasil, 21/06/99, M.D. MOREIRA [printed] \ Erotylidae [printed]”; 1 male (MCNZ, dissected) “Nova Hamburgo, RS, 28/VII/1986 [handwritten], C.J. Becker leg. [printed] \ Col. MCN 238428 [printed]”; 1 female (MCNZ, dissected) “Viamão, RS, Beco do Pesqueiro, AR-Mata Ripária, 30°09′S, 50°58′W, 31.V.2000, A. Bonaldo col. [printed] \ Col. MCN. [printed] 217998 [handwritten]”; 1 female (MCNZ, dissected) “S.F.de Paula, RS (B. dos Bugres), 14.XII.1999, Franceschini, Bonaldo & Silva [printed] \ Col. MCN [printed] 168.713 [handwritten] \ M. tigrinus [handwritten]”; 1 female (MCNZ, dissected) “Tapes, RS (Faz. São Miguel), 17.XII.2003, Equipe Probio col. [printed] \ Col. MCN 225615 [printed]”; 1 female (MCNZ, dissected) “Col. São Pedro de Alcantara, RS, 08/XI/1977 [handwritten], R. Balesteim [? handwritten] \ Col. MCN 238427 [printed]”; 1 female (DZUP, dissected) “Brasil–Paraná, Reserva, 23/III/2007 [printed] \ DZUP 469238 [printed]”; 1 male (MCNZ, dissected) “S.F.de Paula, RS (B. dos Bugres), 14.XII.1999, Franceschini, Bonaldo & Silva [printed] \ Col. MCN [printed] 168.714 [handwritten]”.

**Doubtful identification. **1 female (MCNZ, dissected, pronotum not in good conditions) “Tapes, RS (Faz. São Miguel), 14. V.2003, R.S.de Araujo col. [printed] \ Col. MCN 221798 [printed]”.

### 3.2. Mycotretus centralis Arrow, 1909

**Figure 4:**
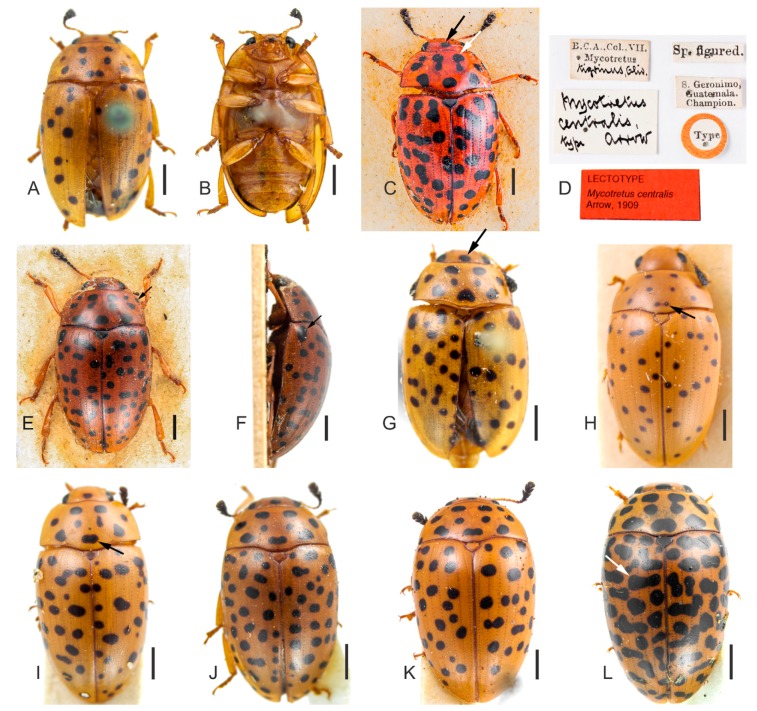
(**A**–**L**) Specimens of *Mycotretus centralis* Arrow, 1909 from different localities: (**A**–**B**) male, dorsal and ventral view, respectively (Carazinho, RS, Brazil). (**C**–**D**) Lectotype (San Jerónimo, Guatemala): (**C**) dorsal view, black arrow showing the major black spot on the disc of head, white arrow showing the two anterior medial spots. (**D**) Labels. (**E**–**F**) A male paralectotype (San Jerónimo, Guatemala): (**E**) dorsal view, arrow showing a spot connected to the lateral pronotal edge, (**F**) lateral view, arrow showing a spot on the humeral angle. (**G**–**L**) Specimens in dorsal view: (**G**) male, arrow showing the major black spot on the disc of head (Campo Bom, RS, Brazil); (**H**) female, arrow showing the medial spots not completely fused (Pitanga, PR, Brazil); (**I**) female, arrow showing the medial spot on the pronotal base (Nova Teutônia, SC, Brazil); (**J**) female (Piraí, Reprêsa Rio Grande, RJ, Brazil); (**K**) female (Piraí, Reprêsa Rio Grande, RJ, Brazil); (**L**) female, arrow showing an elongated spot on the disc of elytra (Pedra Azul, MG, Brazil). Scale bars: **A**–**C**, **E**–**L** = 1 mm.

**Figure 5:**
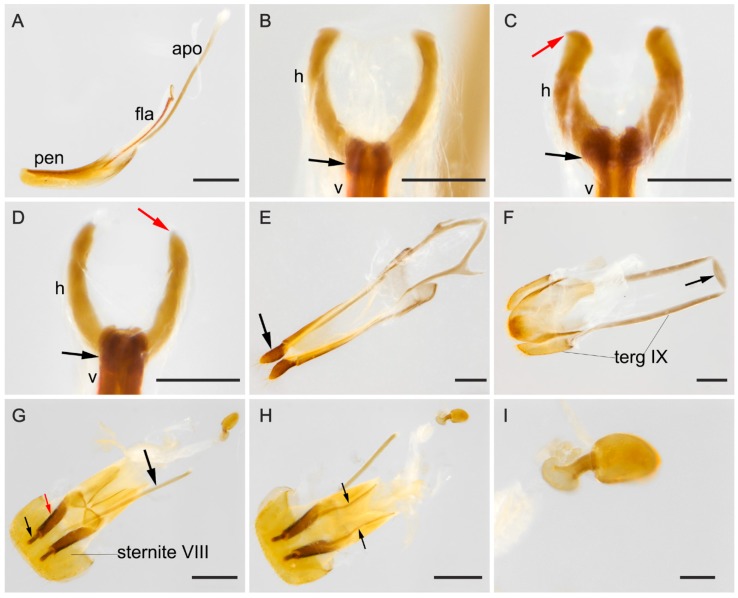
(**A**–**F**) Male abdominal terminalia of *Mycotretus centralis* Arrow, 1909 from different localities: (**A**–**B**,**E**–**F**) Paralectotype from San Jerónimo, Guatemala; (**C**) Campo Bom, RS, Brazil; (**D**) Carazinho, RS, Brazil. (**A**) Lateral view of the aedeagus, showing apophyses (apo), flagellum (fla) and penis (pen). (**B**–**D**) Dorsal view of flagellum showing its head (h) and anterior tip of virga (v); black arrows showing the strongly sclerotized connection between virga and head; red arrow (in C) showing the small and narrow denticle at the outer apical edge of head and (in D) showing the blunt and narrowed tip. (**E**) Tegmen, arrow showing parameres; (**F**) abdominal segments IX–X: laterotergite IX (terg IX), arrow showing the sclerite at the anteroventral edge of segment IX. (**G**–**I**) Female abdominal terminalia of a specimen from Piraí, RJ, Brazil: (**G**) dorsal view, with small black and red arrows showing a gonostylus and a gonocoxite, respectively; big black arrow showing the median strut of sternite VIII; (**H**) ventral view, arrows showing baculi of paraprocts; (**I**) spermatheca. Scale bars: **A**, **G**–**H** = 0.5 mm; **B**–**D**, **I** = 0.1 mm; **E**–**F** = 0.2 mm.

**Figure 6:**
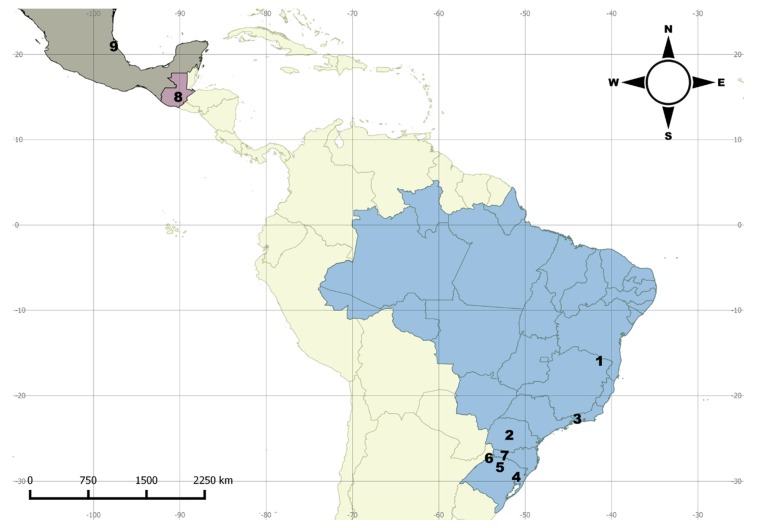
Distribution map for *Mycotretus centralis* Arrow, 1909 species. Brazil (1–7): 1—Pedra Azul (MG); 2—Pitanga (PR); 3—Piraí (RJ); 4—Campo Bom, 5—Carazinho, 6—Derrubadas (RS); 7—Nova Teutônia (SC). Guatemala: 8—San Jerónimo. Mexico: 9—Tuxpan, Vera Cruz.

Figure 4, Figure 5 and Figure 6.

*Mycotretus centralis* Arrow 1909: 196 [9]. Type-locality: San Jerónimo, Guatemala.

**Diagnosis.** Pronotum with black, free and subcircular spots, symmetrically and transversely arranged. Penile flagellum well-developed and slightly elongated, approximately 0.95 × the length of the penis, with a shallow sinuosity and without a membranous portion between the virga and head; in dorsal view, flagellum medially enlarged and slightly sclerotized and, posteriorly, at flagellum head and virga connection, strongly sclerotized. Head of flagellum sclerotized, U-shaped, each branch ending in a blunt and narrowed tip, or forming a shallow, small and narrow denticle at the outer apical edge.

**Redescription.** Length (in mm) = 5.07–6.89 (6.13 ± 0.60, *n* = 9). Body elongate, slightly oval, widest at anterior third of elytra, TL/EW = 1.60–1.77 (1.69 ± 0.06), glabrous and glossy, dorsal and ventral coloration homogeneously yellowish or reddish-brown (Figure 4A–L). Mouthparts of same background color as body, mandible apices blackish and with two teeth; mentum plate pentagonal, with strongly sclerotized margin; antennae yellowish or reddish-brown, last antennomeres blackish. Scutellar shield yellowish, reddish-brown or blackish, glabrous and bearing few punctures. Dorsal coloration: head with one large and subcircular black spot on disc, Figure 4C,G, arrow (specimens from Guatemala with one or two black spots, small, free and close to major spot on disc); pronotum with black, free and subcircular spots (Figure 4) symmetrically and transversely arranged, as follows: three large basal spots (medial one resembling the fusion of the two smallest spots, e.g., Figure 4I, arrow) or with four apparent spots (in this case, medial spots not completely fused, e.g., Figure 4H, arrow); four spots on disc; two or four anterior spots (in the last case, two medial spots bigger than outer ones, Figure 4C, white arrow). In some individuals, spots connected to lateral pronotal edges (Figure 4E, arrow). Elytral coloration with several black, circular and free spots, sparsely distributed. In some individuals, there are elongated spots on each elytron, resembling the fusion of two or three spots, especially on humeral angles. On the disc, somewhat transverse spots can be present (Figure 4L, arrow). 

**Male terminalia.** (Figure 5A–F). Penis (Figure 5A, pen) slightly elongate and curved; basal portion with short sclerotized projection linked to apophyses; internal sac with a well-developed and slightly elongated flagellum (Figure 5A, fla), 0.95 × the length of penis (*n* = 3), with a shallow sinuosity and without a membranous portion between the virga and flagellum head; in dorsal view, flagellum medially enlarged and slightly sclerotized and, posteriorly, at flagellum head and virga connection, strongly sclerotized (Figure 5B–D, black arrow), flagellum head sclerotized (Figure 5B–D), U-shaped, with each branch ending in a blunt and narrowed tip (Figure 5D, red arrow) or, forming a shallow, small and narrow denticle at the outer apical edge (Figure 5C, red arrow). Apophyses (Figure 5A, apo) 1.2 × as long as penis (*n* = 3). Tegmen sclerotized (Figure 5E); parameres reduced and sclerotized, with densely pubescent outgrowths, slightly dilated, narrowed and acute at apex (Figure 5E, arrow). Tergite VIII, sclerotized with sparsely distributed bristles and sternite VIII, slightly sclerotized. Laterotergite IX sclerotized (Figure 5F, terg IX), posteriorly elongated and pubescent, outer contours angulated; anteroventral edge with paired and subparallel lateral struts, connected at its anterior tip, by a small, transverse and slightly sclerotized sclerite (Figure 5F, arrow). Posterior edge of sternite IX, sclerotized, undivided, outer contour rounded; anteriorly membranous. Tergite X, sclerotized, anterior edge with sparsely distributed bristles. 

**Female terminalia.** (Figure 5G–I). Gonostyli and gonocoxites strongly sclerotized (Figure 5G, black and red arrows, respectively, under sternite VIII), baculi of paraprocts sclerotized and sinuous (Figure 5H, arrows); spermatheca oval and sclerotized (Figure 5I). Tergite VIII sclerotized and sternite VIII with conspicuous median strut (Figure 5G, sternite VIII, big black arrow).

**Distribution**. North and Central America, South and Southeast Brazil (Figure 6). 

**Remarks**. (1) *Mycotretus centralis* has a disjunct geographical distribution (Figure 6). See Discussion. (2) The flagellum head of the dissected Brazilian *M. centralis* (Figure 5C–D) is more sclerotized and thicker than a specimen from Guatemala (Figure 5B). (3) Interestingly, based on the pronotal color pattern of *M. centralis* described here, the specimen figured by Gorham as an example of *M. tigrinus* (see Gorham [1], Tab. III. Figure 9) is probably *M. centralis.* (4) A female from Derrubadas (RS, Brazil) seems to have the usual pronotal coloration of *M. centralis*. However, as the specimen may be a teneral, we considered it a doubtful identification. 

**Material examined. Lectotype of *M. centralis *Arrow, 1909, here designated **(BMNH) (Figure 4C–D) “B.C.A., Col., VII. Mycotretus [printed] tigrinus, Oliv. [handwritten] \ Mycotretus centralis type arrow [handwritten] \ Sp. figured. [printed] \ S. Geronimo, Guatemala. Champion. [printed] \ Type [printed, disc-shaped label with red contour] \ LECTOTYPE *Mycotretus centralis *Arrow, 1909 [printed, red label]”; 1 male (BMNH, dissected) “S. Geronimo, Guatemala. Champion. [printed] \ B.C.A.,Col.,VII. Mycotretus [printed] tigrinus, Oliv. [handwritten] \ PARALECTOTYPE *Mycotretus centralis *Arrow, 1909 [printed, yellow label]”; 2 specimens (BMNH, on the same card) “S. Geronimo, Guatemala. Champion. [printed] \ B.C.A.,Col.,VII. Mycotretus [printed] tigrinus, Oliv. [handwritten] \ PARALECTOTYPE *Mycotretus centralis *Arrow, 1909 [printed, yellow label]”; 1 specimen (BMNH) “Toxpam [printed] \ Mexico. Salle coll. [printed] \ 2398 [printed] \ B.C.A.,Col.,VII. Mycotretus [printed] tigrinus, Oliv. [handwritten] \ PARALECTOTYPE *Mycotretus centralis *Arrow, 1909 [printed, yellow label]”; 1 female (DZUP, dissected) “Coleção M. Alvarenga [printed] \ REPRÊSA RIO GRANDE, Guanabara BRASIL [printed] IX. 1964 [handwritten], F.M. Oliveira [printed] \ DZUP 371269 [printed]”; 1 female (DZUP, dissected) “Coleção M. Alvarenga [printed] \ REPRÊSA RIO GRANDE, Guanabara BRASIL [printed] X. 1967 [handwritten], F.M. Oliveira [printed] \ DZUP 371268 [printed]”; 1 female (DZUP, dissected) “Coleção M. Alvarenga [printed] \ PEDRA AZUL, 700M, M. Gerais, Brasil, XI.1972, Seabra & Oliveira [printed] \ DZUP 371277 [printed]”; 1 female (DZUP, dissected) “4 X [handwritten] 194[printed] 7[handwritten], Brasilien, Nova Teutonia, 27°11′B, 52°23′L, Fritz Plaumann, 300 b[?] 500 m [printed] \ DZUP 125457 [printed]”; 1 male (MCNZ, dissected) “Campo Bom, RS, 29/ IV/ [handwritten] 19 [printed] 88 [handwritten], C.J. Becker leg. [printed] \ Col. MCN [printed] 150.874 [handwritten]”; 1 male (MCNZ, dissected) “Carazinha, RS, 10/XI/ [handwritten] 19 [printed] 79 [handwritten], A. Lise leg. [printed] \ Col. MCN 28.682 [handwritten]”; 1 female (DZUP, dissected) “PITANGA, 24°41, 51°46, 700 m [printed] \ MAERZ 1963, F. Plaumann [printed] \ DZUP 125578 [printed]”; 

**Doubtful identification.** 1 female (MCNZ, dissected) “27°14′14.7″ S, 53°58′46.0″ W [printed] \ Derrubadas, RS (Pq. Est. Turvo), 30.X.2003, L. Heydrich col. [printed] \ Col. MCN 227465 [printed]”.

### 3.3. Mycotretus tigrinoides Mader, 1942

**Figure 7:**
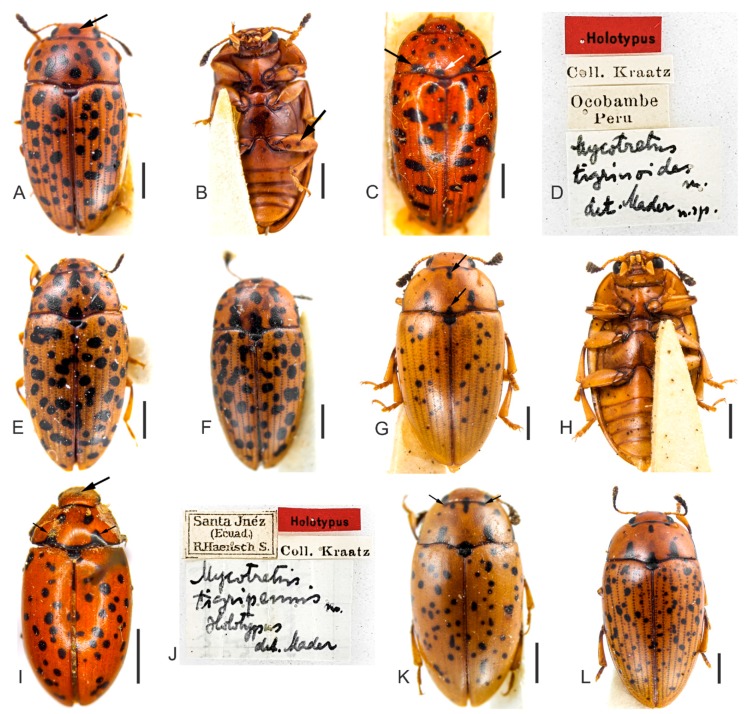
(**A**–**F**) Specimens of *Mycotretus tigrinoides* Mader, 1942 from different localities: (**A**–**B**) male, dorsal and ventral view, respectively (Tucuruí, PA, Brazil): (**A**) arrow showing the major black spot on the disc of head, (**B**) arrow showing a subcircular spot on metafemur. (**C**–**D**) Holotype (Ocobamba, Peru): (**C**) dorsal view, black arrows showing the two lateral spots on the basal pronotal edge and white arrow showing the inner spot. (**D**) Labels. (**E**) Female (Brasília, DF, Brazil); (**F**) male (Sinop, MT, Brazil). (**G**–**L**) Specimens of *Mycotretus tigripennis* Mader, 1942 from different localities: (**G**–**H**) male, dorsal and ventral view, respectively (Piraí, Reprêsa Rio Grande, RJ, Brazil): (**G**) arrows showing elongated and medial spots between two lateral tooth-like spots on pronotum. (**I**–**J**) Holotype (Santa Inéz, Ecuador): (**I**) big arrow showing one black spot on the disc of head, small arrows showing two lateral tooth-like spots on basal pronotal edge. (**J**) Labels. (**K**) Female (Piraí, Reprêsa Rio Grande, RJ, Brazil), arrows showing two lateral tooth-like spots on anterior pronotal edge. (**L**) Female (Piraí, Reprêsa Rio Grande, RJ, Brazil). Scale bars: **A**–**C**, **E**–**H**, **K**–**L** = 1 mm; **I** = 2 mm.

**Figure 8:**
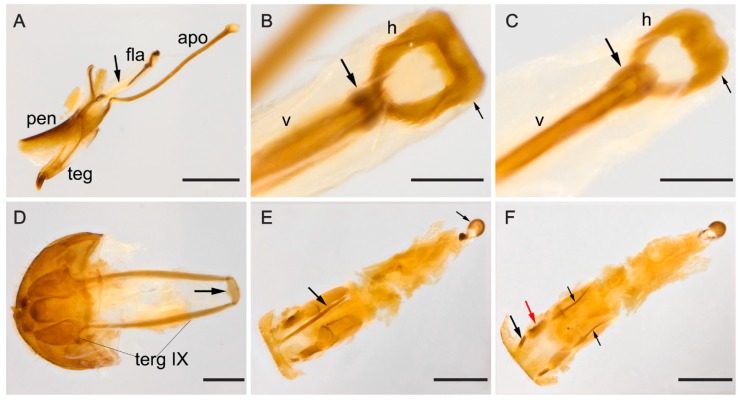
(**A**–**D**) Male abdominal terminalia of *Mycotretus tigrinoides* Mader, 1942 from two localities: (**A**–**B**,**D**) Sinop, MT, Brazil; (**C**) Tucuruí, PA, Brazil. (**A**) Lateral view of the aedeagus, showing apophyses (apo), flagellum (fla), penis (pen) and tegmen (teg); arrow showing a medial desclerotization in flagellum. (**B**–**C**) Flagellum dorsal view, showing: head (h) and anterior tip of virga (v); black arrow showing the sclerotization between virga and head, small arrow showing the outer anterior contour forming a right angle. (**D**) Abdominal segments VIII–X: laterotergite IX (terg IX), arrow showing the sclerite at the anteroventral edge of segment IX. (**E**–**F**) Female abdominal terminalia of a specimen from Brasília, DF, Brazil: (**E**) dorsal view, big arrow showing the median strut of sternite VIII, small arrow showing the spermatheca; (**F**) ventral view, small black and red arrows showing a gonostylus and a gonocoxite, respectively; small arrows showing baculi of paraprocts. Scale bars: **A**, **E**–**F** = 0.5 mm; **B**–**C** = 0.1 mm; **D** = 0.2 mm.

Figure 7A–F, Figure 8 and Figure 10 (localities 1–5, map).

*Mycotretus tigrinoides* Mader 1842: 174, 196 [10]. Type locality: Ocobamba, Peru.

**Diagnosis.** Pronotum with sparsely, black, free, subcircular elongated spots, asymmetrically arranged. In most specimens, there are three spots with no definite shape close to basal pronotal edge; lateral spots more elongated than inner ones. Elytral spots somewhat transverse, forming true transverse spots in some specimens. Penile flagellum well-developed, sclerotized and slightly elongated, approximately 0.97 × the length of the penis, shallowly sinuous, with prominent medial desclerotization (absent in *M. tigripennis*). Anterior tip of virga with prominent and strong sclerotization (absent in *M. tigripennis*). The flagellum of *M. tigrinoides* is narrower dorsally than that of *M. tigripennis*. Head of flagellum sclerotized and subpentagonal, outer anterior contours forming a right angle. 

**Redescription**. Length (in mm) = 3.95–5.46 (4.85 ± 0.51, *n* = 13). Very similar to *M. tigripennis*. Body elongate, subparallel-sided, widest at the anterior third of elytra, TL/EW = 1.80–1.91 (1.86 ± 0.03), glabrous and glossy; dorsal and ventral coloration (Figure 7A–F) reddish-brown, with mouthparts and first antennomeres yellowish to reddish-brown; legs yellowish to reddish-brown with coxae and tibiae partially blackish in some individuals; mandible apices and last antennomeres blackish; mentum plate pentagonal, with strongly sclerotized margin. Venter usually with black and subcircular spots on prosternum, meso- and meta-ventrite, abdomen and legs (Figure 7B, arrow). Scutellar shield reddish-brown or blackish, glabrous and bearing few punctures. Dorsal coloration: head with one large and subcircular black spot on disc, Figure 7A, arrow, (specimen from Brasilia, DF, Brazil with three black, small and free spots close to major spot on disc); pronotum with sparsely, black, free, subcircular and elongated spots, asymmetrically arranged. In most specimens, three spots with no definite shape close to basal edge, lateral spots more elongated (Figure 7C, black arrows) than inner ones (Figure 7C, white arrow). Elytral coloration with several black, free and subcircular spots, sparsely and asymmetrically distributed. Elytral spots somewhat transverse, forming true transverse spots in some specimens (Figure 7C,E). 

**Male terminalia.** (Figure 8A–D). Penis (Figure 8A, pen) slightly elongated and curved; basal portion with short sclerotized projection linked to apophyses; internal sac with well-developed, sclerotized and slightly elongated flagellum (Figure 8A, fla), 0.97 × the length of penis (*n* = 2), shallowly sinuous, with prominent medial desclerotization (Figure 8A, arrow, absent in *M. tigripennis*). Anterior tip of virga with prominent and strong sclerotization (Figure 8B,C, arrow) absent in *M. tigripennis*). Flagellum of *M. tigrinoides* narrowed dorsally compared to that of *M. tigripennis* (Figure 8B,C). Head of flagellum (Figure 8B,C) sclerotized and subpentagonal, outer anterior contours forming a right angle (Figure 8B,C, small arrow) and anterior edge enlarged, compared to posterior one. Apophyses (Figure 8A, apo) 1.58 × as long as penis (*n* = 2). Tegmen sclerotized (Figure 8A, teg); parameres reduced and sclerotized, with densely pubescent outgrowths, slightly dilated and acute at apex. Tergite VIII, sclerotized with sparsely distributed bristles. Sternite VIII slightly sclerotized. Laterotergite IX sclerotized (Figure 8D, terg IX), posteriorly elongated and pubescent, outer contours angulated; anteroventral edge with paired and subparallel lateral struts, connected at its anterior tip by small, transverse, slightly sclerotized sclerite (Figure 8D, arrow). Posterior edge of sternite IX sclerotized, undivided, outer contour rounded; anteriorly membranous. Tergite X sclerotized; anterior edge truncate, with sparsely distributed bristles. 

**Female terminalia.** (Figure 8E,F). Gonostyli and gonocoxites strongly sclerotized (Figure 8F, black and red arrows, respectively), baculi of paraprocts sclerotized and slightly arcuate (Figure 8F, black small arrows); spermatheca oval and sclerotized (Figure 8E, black small arrow). Tergite VIII sclerotized and sternite VIII with conspicuous median strut (Figure 8E, big arrow).

**Distribution.** Peru, North and Central Brazil (Figure 10, localities 1–5, black numerals).

**Remarks**. Both *M. tigrinoides* and *M. tigripennis* (redescribed below) were described based only on their primary types (Figure 7C,D,I,J, respectively), which were not dissected here. However, based on the original descriptions and images of holotypes, we identified the specimens examined by us as intraspecific variations.

**Material examined**. 1 specimen (SDEI) “Holotypus [printed, Red label] \ Coll. Kraatz [printed] \ Ocobambe Peru [printed] \Mycotretus tigrinoides [handwritten] Ma. [?] det. Mader n.sp. [handwritten]”; 1 male (DZUP, dissected) “Coleção M. Alvarenga [printed] \ SINOP, M. Grosso, Brasil, X. 1974, M. Alvarenga [printed] \ Lat 12° 31′ S, Lon 55° 37′ W [printed] \ DZUP 126115 [printed]”; 1 female (CAMB, dissected) “Brasília, DF – BRASIL [printed] XI [handwritten] 2000, Col. N. Degallier [printed] \ Coleção A.M. BELLO [printed]”; 1 male (MNRJ, dissected) “Coleção M. Alvarenga [printed] \ Tucuruí, Pará, Brasil, XI-1985, N. Degallier [handwritten] \ AI [printed]”; 1 specimen (DZUP) “Coleção M. Alvarenga [printed] \ SINOP, M. Grosso, Brasil, X. 1974, M. Alvarenga [printed] \ Lat 12° 31′ S, Lon 55° 37′ W [printed] \ DZUP 126110 [printed]”; 1 specimen (DZUP) “Coleção M. Alvarenga [printed] \ SINOP, M. Grosso, Brasil, X. 1974, M. Alvarenga [printed] \ Lat 12° 31′ S, Lon 55° 37′ W [printed] \ DZUP 126111 [printed]”; 1 specimen (DZUP) “Coleção M. Alvarenga [printed] \ SINOP, M. Grosso, Brasil, X. 1974, M. Alvarenga [printed] \ Lat 12°31′ S, Lon 55° 37′ W [printed] \ DZUP 126112 [printed]”; 1 specimen (DZUP) “Coleção M. Alvarenga [printed] \ SINOP, M. Grosso, Brasil, X. 1974, M. Alvarenga [printed] \ Lat 12° 31′ S, Lon 55°37′ W [printed] \ DZUP 126113 [printed]”; 1 specimen (DZUP) “Coleção M. Alvarenga [printed] \ V. VERA, M. Grosso, Brasil, X. 1973, M. Alvarenga [printed] \ Lon 55°36′ W, Lat 12° 46′ S [printed] \ DZUP 126117 [printed]”; 1 specimen (DZUP) “Coleção M. Alvarenga [printed] \ SINOP, M. Grosso, Brasil, X. 1974, M. Alvarenga [printed] \ Lat 12°31’ S, Lon 55°37′ W [printed] \ DZUP 126114 [printed]”; 1 specimen (DZUP) “Coleção M. Alvarenga [printed] \ V. VERA, M. Grosso, Brasil, X. 1973, M. Alvarenga [printed] \ Lon 55°36′ W, Lat 12°46′ S [printed] \ DZUP 126118 [printed]”; 1 specimen (DZUP) “Coleção M. Alvarenga [printed] \ SINOP, M. Grosso, Brasil, X. 1974, M. Alvarenga [printed] \ Lat 12°31′ S, Lon 55°37′ W [printed] \ DZUP 126119 [printed]”.

### 3.4. Mycotretus tigripennis Mader, 1942

**Figure 9:**
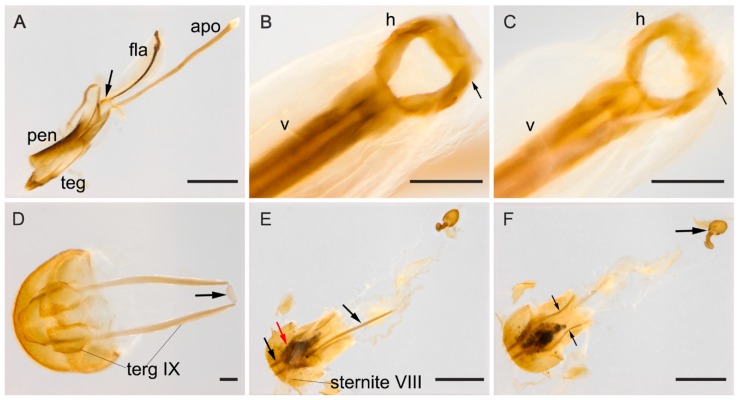
(**A**–**D**) Male abdominal terminalia of two specimens of *Mycotretus tigripennis* Mader, 1942 from Piraí, RJ, Brazil: (**A**) lateral view of the aedeagus, showing apophyses (apo), flagellum (fla), penis (pen) and tegmen (teg); arrow showing a shallow desclerotization posteriorly. (**B**–**C**) Flagellum dorsal view, showing: head (h) and anterior tip of virga (v); arrow showing the outer anterior contour with an acute angle. (**D**) Abdominal segments VIII–X: laterotergite IX (terg IX), arrow showing the sclerite at the anteroventral edge of segment IX. (**E**–**F**) Female abdominal terminalia of a specimen from Piraí, RJ, Brazil: (**E**) dorsal view, big arrow showing the median strut of sternite VIII, small black and red arrows showing a gonostylus and a gonocoxite, respectively; (**F**) ventral view, small arrows showing baculi of paraprocts and big arrow showing the spermatheca. Scale bars: **A**, **E**–**F** = 0.5 mm; **B**–**D** = 0.1 mm.

**Figure 10:**
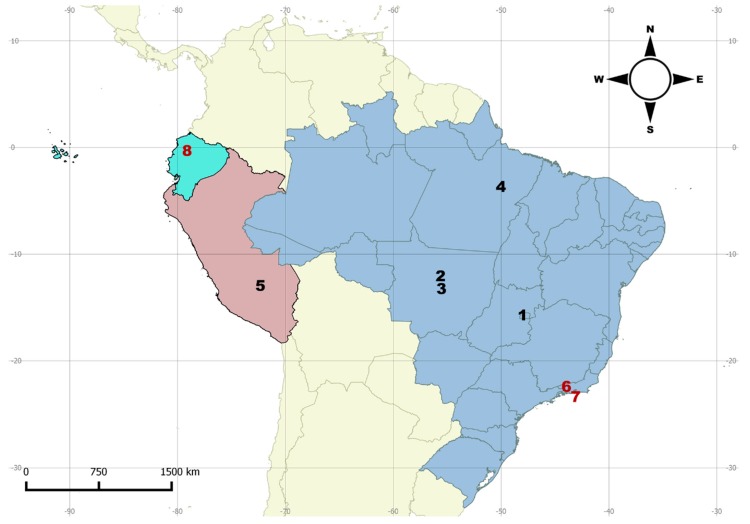
Distribution map for *Mycotretus tigrinoides* Mader, 1942 and *Mycotretus tigripennis* Mader, 1942 species. *Mycotretus tigrinoides* (1–5, black numerals): Brazil (1–4): 1—Brasília (DF); 2—Sinop (MT); 3—Vera (MT); 4—Tucuruí (PA). Peru: 5—Ocobamba. *Mycotretus tigripennis* (6–8, reddish numerals): Brazil (6–7): 6—Piraí (RJ); 7—Rio de Janeiro, Floresta da Tijuca (RJ). Ecuador: 8—Santa Inés (Ecuador).

Figure 7G–L, Figure 9 and Figure 10 (localities 6–8, map).

*Mycotretus tigripennis* Mader 1942: 174, 197 [10]. Type locality: Santa Inéz, Ecuador.

**Diagnosis.** Posterior and anterior edge of pronotum with a black, shallow and transverse mark, from which two lateral tooth-like spots arise, sometimes with a medial elongated spot; subcircular pronotal spots can be present laterally or on the disc. Elytral coloration with several black, free, subcircular or longitudinal spots, sparsely distributed (some of which are apparently fused). Penile flagellum well-developed and slightly elongated, approximately 1.34 × the length of the penis, anteriorly arcuate and with a shallow desclerotization posteriorly (absent in *M. tigrinoides*). Flagellum of *M. tigripennis* dorsally broader than that of *M. tigrinoides*. Head of flagellum sclerotized and subpentagonal, with outer anterior contours forming an acute angle.

**Redescription**. Length (in mm) = 4.52–6.25 (5.52 ± 0.49, n = 17). Body elongate, widest at the anterior third of elytra, TL/EW = 1.76–1.86 (1.81 ± 0.02), glabrous and glossy; dorsal and ventral coloration (Figure 7G–L) homogeneously yellowish or reddish-brown; mouthparts and first antennomeres yellowish to reddish-brown; legs yellowish to reddish-brown, with coxae and tibiae partially blackish in some individuals; mandible apices partially black and last antennomeres blackish; mentum plate pentagonal, with a strongly sclerotized margin. Venter usually black with subcircular spots. Scutellar shield blackish, glabrous and bearing few punctures. Dorsal coloration: head with no spots or with one large and subcircular black spot on the disc (Figure 7I, arrow); posterior and anterior edge of pronotum with black, shallow and transverse marks, from which two lateral tooth-like spots arise (Figure 7I,K, small arrows), sometimes with in between elongated and medial spots (Figure 7G, arrow); sometimes with subcircular pronotal spots laterally or on the disc. Elytral coloration with several black, free, subcircular or longitudinal spots, sparsely distributed (some apparently fused). 

**Male terminalia.** (Figure 9A–D). Penis (Figure 9A, pen) slightly elongated and curved; basal portion with short sclerotized projection linked to apophyses; internal sac with well-developed and slightly elongated flagellum (Figure 9A, fla), 1.34 × the length of penis (*n* = 2), anteriorly arcuate and with shallow desclerotization posteriorly (Figure 9A, arrow, absent in *M. tigrinoides*). Flagellum of *M. tigripennis* dorsally broader than that of *M. tigrinoides* (Figure 9B,C). Head of flagellum (Figure 9B,C) sclerotized and subpentagonal; outer anterior contours forming an acute angle (Figure 9B,C, arrow), anterior edge enlarged compared to posterior edge. Apophyses (Figure 9A, apo) 1.51 × as long as penis (*n* = 2). Tegmen sclerotized (Figure 9A, teg); parameres reduced and sclerotized, with densely pubescent outgrowths, slightly dilated, narrowed and acute at apex. Tergite VIII, sclerotized with sparsely distributed bristles. Sternite VIII slightly sclerotized. Laterotergite IX sclerotized (Figure 9D, terg IX), posteriorly elongated and pubescent; outer contours angulated; anteroventral edge with paired and subparallel lateral struts, connected at its anterior tip by small, transverse, slightly sclerotized sclerite (Figure 9D, arrow). Posterior edge of sternite IX sclerotized, undivided; outer contour rounded; anteriorly membranous. Tergite X sclerotized; anterior edge truncate, with sparsely distributed bristles.

**Female terminalia.** (Figure 9E,F). Gonostyli and gonocoxites strongly sclerotized (Figure 9E, black and red arrows, respectively, under sternite VIII); baculi of paraprocts sclerotized and arcuate (Figure 9F, black small arrows); spermatheca oval and sclerotized (Figure 9F, big black arrow). Tergite VIII sclerotized and sternite VIII with conspicuous median strut (Figure 9E, big arrow).

**Distribution**. Ecuador (Santa Inéz) and Southeast Brazil (Figure 10, localities 6–8, reddish numerals). 

**Remarks**. See the above remarks of *M. tigrinoides* concerning the identification of examined specimens of *M. tigripennis*. The specimens identified here as *M. tigripennis* (from Rio de Janeiro, Brazil) are far away from the type locality (Santa Inés, Ecuador) and we thought that it would be a new species at first. Although that remains a possibility, until other populations are studied, we prefer to consider these Brazilian specimens as intraspecific variation of *M. tigripennis*.

**Material examined**. 1 specimen (SDEI) “Holotypus [printed, red label] \ Santa Jnéz (Ecuad.) R. Haensch S. [printed] \ Coll. Kraatz [printed] \ Mycotretus tigripennis [handwritten] Ma. [?] Holotypus det. Mader [handwritten]”; 1 male (DZUP, dissected) “REPRÊSA RIO GRANDE, Guanabara BRASIL [printed] XII. 1960 [handwritten], F.M. Oliveira [printed] \ DZUP 127806 [printed]”; 1 female (DZUP, dissected) “Coleção M. Alvarenga [printed] \ REPRÊSA RIO GRANDE, Guanabara BRASIL [printed] II. 1967 [handwritten], F.M. Oliveira [printed] \ DZUP 127795 [printed]”; 1 female (DZUP, dissected) “Coleção M. Alvarenga [printed] \ REPRÊSA RIO GRANDE, Guanabara BRASIL [printed] III. 1967 [handwritten], F.M. Oliveira [printed] \ DZUP 127804 [printed]”; 1 female (MNRJ, dissected) “Coleção M. Alvarenga [printed] \ REPRÊSA RIO GRANDE, Guanabara BRASIL [printed] III. 1964 [handwritten], F.M. Oliveira [printed]”; 1 female (DZUP, dissected) “Coleção M. Alvarenga [printed] \ REPRÊSA RIO GRANDE, Guanabara BRASIL [printed] XII. 1960 [handwritten], F.M. Oliveira [printed] \ DZUP 127808 [printed]”; 1 female (DZUP, dissected) “Coleção M. Alvarenga [printed] \ REPRÊSA RIO GRANDE, Guanabara BRASIL [printed] III. 1967 [handwritten], F.M. Oliveira [printed] \ DZUP 127789 [printed]”; 1 male (DZUP, dissected) “Coleção M. Alvarenga [printed] \ REPRÊSA RIO GRANDE, Guanabara BRASIL [printed] III. 1967 [handwritten], F.M. Oliveira [printed] \ DZUP 127805 [printed]”; 1 specimen (DZUP) “Coleção M. Alvarenga [printed] \ REPRÊSA RIO GRANDE, Guanabara BRASIL [printed] VIII. 1969 [handwritten], F.M. Oliveira [printed] \ DZUP 127824 [printed]”; 1 specimen (DZUP) “Coleção M. Alvarenga [printed] \ REPRÊSA RIO GRANDE, Guanabara BRASIL [printed] IX. 1964 [handwritten], F.M. Oliveira [printed] \ DZUP 127796 [printed]”; 1 specimen (DZUP) “REPRÊSA RIO GRANDE, Guanabara BRASIL [printed] II. 1967 [handwritten], F.M. Oliveira [printed] \ DZUP 127797 [printed]”; 1 specimen (DZUP) “Coleção M. Alvarenga [printed] \ REPRÊSA RIO GRANDE, Guanabara BRASIL [printed] III. 1968 [handwritten], F.M. Oliveira [printed] \ DZUP 127801 [printed]”; 1 specimen (DZUP) “Coleção M. Alvarenga [printed] \ REPRÊSA RIO GRANDE, Guanabara BRASIL [printed] II. 1967 [handwritten], F.M. Oliveira [printed] \ DZUP 127794 [printed]”; 1 specimen (DZUP) “Coleção M. Alvarenga [printed] \ REPRÊSA RIO GRANDE, Guanabara BRASIL [printed] XII. 1960 [handwritten], F.M. Oliveira [printed] \ DZUP 127809 [printed]”; 1 specimen (DZUP) “Coleção M. Alvarenga [printed] \ FLORESTA da TIJUCA, D. Federal, Brasil [printed] I. 1961 [handwritten], C.A.C. Seabra\ DZUP 127830 [printed]”; 1 specimen (DZUP) “Coleção M. Alvarenga [printed] \ FLORESTA da TIJUCA, D. Federal, Brasil [printed] I. 1961 [handwritten], C.A.C. Seabra\ DZUP 127828 [printed]”; 1 specimen (DZUP) “Coleção M. Alvarenga [printed] \ FLORESTA da TIJUCA, D. Federal, Brasil [printed] I. 1961 [handwritten], C.A.C. Seabra\ DZUP 127829 [printed]”; 1 specimen (DZUP) “Coleção M. Alvarenga [printed] \ REPRÊSA RIO GRANDE, Guanabara BRASIL [printed] IX. 1969 [handwritten], F.M. Oliveira [printed] \ DZUP 126096 [printed]”.

### 3.5. Mycotretus prioteloides Mader, 1942

**Figure 11:**
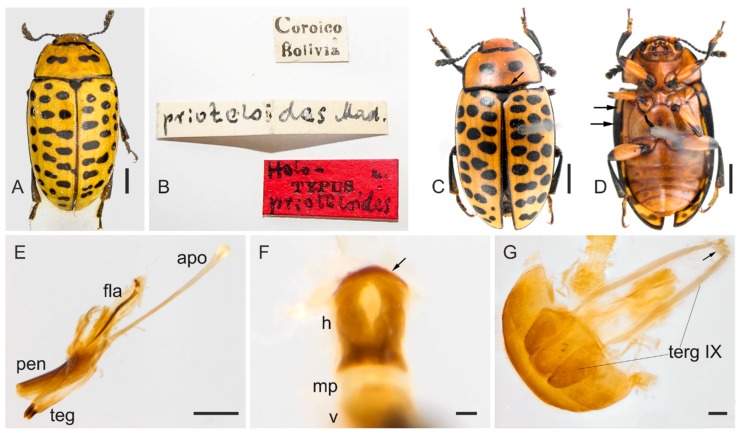
(**A**–**D**) Specimens of *Mycotretus prioteloides* Mader, 1942: (**A**–**B**) holotype (Coroico, Bolivia): (**A**) dorsal view, black arrow showing the transverse black mark at the anterior pronotal edge. (**B**) Labels. (**C**–**G**) Male (Torentoy Canyon, Base Machu Picchu, Peru): (**C**–**D**) dorsal and ventral view, respectively, (**E**–**G**) genitalia. (**C**) Arrow showing the black mark at the posterior pronotal edge; (**D**) arrows showing the elytral epipleuron partially black and yellowish; (**E**) lateral view of the aedeagus, showing apophyses (apo), flagellum (fla), penis (pen) and tegmen (teg); (**F**) dorsal view of the flagellum, showing head (h), anterior tip of virga (v) and the membranous portion (mp), arrow showing conspicuously convex anterior edge; (**G**) abdominal segments VIII–X: laterotergite IX (terg IX), arrow showing the sclerite at the anteroventral edge of segment IX. Scale bars: **A**, **C**–**D** = 1 mm; **E** = 0.5 mm; **F**–**G** = 0.1 mm.

**Figure 12:**
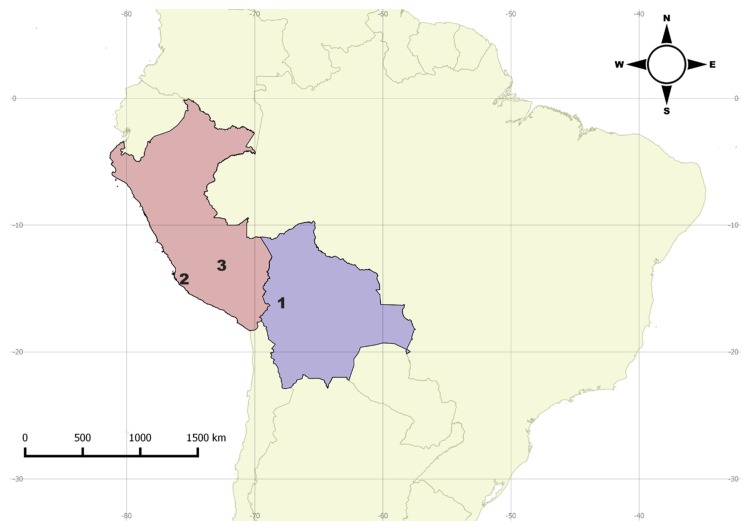
Distribution map for *Mycotretus prioteloides* Mader, 1942. Bolivia: 1—Coroico. Peru: 2—Calango; 3—Machu Picchu.

Figure 11 and Figure 12.

*Mycotretus prioteloides* Mader 1942: 174, 196 [10]. Type locality: Coroico, Bolivia. 

**Diagnosis.** Pronotal edges black, with a shallow and transverse black mark at posterior or anterior edges; pronotal portion with two transverse or subcircular black spots on the disc. Outer edges of pronotum and mesal sutural edge of elytra with black outline. Elytral coloration with several black, subcircular and transverse free spots. Penile flagellum well-developed and slightly elongated, approximately 0.8 × the length of penis, slightly sinuous, with a membranous portion between its virga and head. Head of flagellum sclerotized, slightly elongated and, unlike in *M. tigrinus*, conspicuously convex anteriorly; inner outline somewhat more separated than in *M. tigrinus*.

**Redescription**. Length (in mm) = 5.75–7 (6.29 ± 0.63, *n* = 3). Body elongate, widest at anterior third of elytra, TL/EW = 1.75–1.91 (1.83 ± 0.08) (measurements based on examined specimen and in Mader’s original description based on holotype and one paratype), glabrous and glossy, dorsal and ventral coloration homogeneously yellowish or reddish-brown (Figure 11A,C,D). Mouthparts reddish-brown with outer contour of last maxillary and labial palpomeres yellowish; mentum plate pentagonal, with strongly sclerotized margin; mandible apices and antennomeres 2–11 blackish (scape reddish-brown). Trochanters, apical portion of femora, tibiae and dorsum of tarsi blackish; tarsi (ventrally) and claws yellowish to reddish-brown. Elytral epipleuron partially black and yellowish (Figure 11D, arrows). Scutellar shield blackish, glabrous and bearing few punctures. Dorsal coloration: head without spots; pronotal edges black, with a shallow and transverse black mark on the anterior (Figure 11A, arrow) and posterior edge (Figure 11C, arrow); disc of pronotum with two transverse (Figure 11A) or subcircular (Figure 11C) black spots. Outer edges of pronotum and mesal sutural edge of elytra with black outline. Elytral coloration with several black, subcircular and transverse free spots. 

**Male terminalia.** (Figure 11E–G). Penis (Figure 11E, pen) slightly elongated and curved; basal portion with short sclerotized projection linked to apophyses; internal sac with well-developed and slightly elongated flagellum (Figure 11E, fla), 0.8 × the length of penis (*n* = 1), slightly sinuous, with a membranous portion between the virga and head (Figure 11F, arrow). Head of flagellum (Figure 11F) sclerotized, slightly elongated, and unlike in *M. tigrinus*, conspicuously convex anteriorly (Figure 11E, small arrow); inner outline somewhat more separated than in *M. tigrinus*. Apophyses (Figure 11E, apo) 1.3 × as long as penis. Tegmen sclerotized (Figure 11A, teg); parameres reduced and sclerotized, with densely pubescent outgrowths, slightly dilated, narrowed and acute at apex. Tergite VIII, sclerotized with sparsely distributed bristles. Sternite VIII slightly sclerotized. Laterotergite IX sclerotized (Figure 11G, terg IX), posteriorly elongated and pubescent, outer contours angulated; anteroventral edge with paired and subparallel lateral struts connected at its anterior tip by small, transverse, slightly sclerotized sclerite (Figure 11G, arrow). Posterior edge of sternite IX sclerotized, undivided; anteriorly membranous. Tergite X sclerotized, anterior edge with sparsely distributed bristles.

**Female terminalia**. Unknown.

**Distribution**. Bolivia (Coroico), Peru (Calango, Machu Picchu) (Figure 12). 

**Remarks**. Although *M. prioteloides* is remarkably distinct from the other *Mycotretus* studied here, its male genitalia resembles that of *M. tigrinus* and, apparently, both species are closely related. 

**Material examined**. 1 specimen (NMBS) “Coroico Bolivia [printed] \ prioteloides Mad. [handwritten] \ holo- [handwritten], TYPUS [printed], prioteloides [handwritten] [red label]”; 1 male (MNRJ, dissected) “Coleção M. Alvarenga [printed] \ Homeotipo [printed, red label] \ Torentoy Canyon (Base Machu – Pichu), 2000–2000 m––PERU, VI–VII. 964 B. Malkin [printed] \ Comparado com tipo [printed], Mycotretus prioteloides Mader, 1942 [handwritten], M. alvarenga det. 1971 [printed] \ 1747 [printed]”.

## 4. Discussion

The disjunct geographical distribution of *M. centralis* and *M. tigripennis* are not exceptions in the genus. For instance, such a distribution was also observed in *M. chilensis* Crotch, 1876 and *M. trifasciatus* Guérin, 1956 [3]. In the latter two cases, the majority of records are from the Atlantic Forest biome and a single outlier for each species (considered by the authors as doubtful records) leads their distribution to be interpreted as disjunct. Here we point out two other species with disjunct distributions: *M. centralis* and *M. tigripennis*. In *M. centralis,* there are two records from North and Central America (Figure 6) and the others are from the Atlantic Forest in Brazil. In *M. tigripennis,* there is a record from northwestern South America and the others are from the Brazilian Atlantic Forest (Figure 10, localities 6–8). Other examples of disjunct distribution in *Mycotretus* are mentioned by Alvarenga [2], e.g., *M. nigroterminatus* Lacordaire, 1842 (Colombia and Southeast Brazil), *M. coccineus* Lacordaire, 1842 (north Neotropical region and Southeast Brazil) and *M. sanguineus* (Duponchel, 1825) (Colombia and Southeast Brazil). The cases of disjunct distribution of *Mycotretus* species have been accumulating and now we cannot simply attribute these to collection gaps a priori. It is important to gather host fungi data and compare host use of these species between occurrence areas separated by great gaps, which would be a sign of isolation of well separated populations. On the other hand, recently acquired or compiled data have shown that some *Mycotretus* species are broadly distributed and extend almost continuously along extensive areas, from the southern to the northern Neotropical region, as in the case of *M. tigrinus*, in which the accumulation of records revealed areas where the species is less frequently collected, breaking the disjunct distribution pattern. Neotropical erotylids have been barely collected and studied, especially those from the southern neotropics, and thousands of unidentified specimens housed in scientific collections remain to be studied.

Three species studied here are recorded for the first time from Brazil: *M. centralis*, *M. tigrinoides* and *M. tigripennis*. The former, *M. centralis*, is sympatric with *M. tigrinus* in Nova Teutônia (SC) and with *M. tigripennis* in Piraí (RJ). Sympatry is an event already verified in *Mycotretus* [3,4,24] and “syntopy”, a particular case of sympatry in which two or more related species occupy the same microhabitat in the same locality, was already reported for two species, *M. chilensis* Crotch, 1876 and *M. trifasciatus* Guérin, 1956 [3]. In this context, further ecological studies shall evaluate whether *M. centralis*, *M. tigrinus* and *M. tigripennis* share the same microhabitats (i.e., the same host fungi) or not.

The morphology of the penile flagellum of *M. tigrinus* and *M. prioteloides*, with a membranous portion between its head and virga and an elongated head with the inner contours slightly separated, resembles that of other species dissected by us for forthcoming work, e.g., *M. sallei* Crotch, 1876, *M. lesueuri* (Chevrolat, 1835), *M. sobrinus* (Guérin-Méneville, 1841), *M. spadiceus* Gorham, 1888 and *M. savignyi* Lacordaire, 1842 (personal observation). Considering these morphological similarities, ongoing work will evaluate whether these species are phylogenetically closely related or are additional synonyms of *M. tigrinus*. However, the coloration of the aforementioned species differs greatly from that of *M. tigrinus*. The coloration of *M. lesueuri*, *M. sobrinus* and *M. savignyi* is homogeneously red, with black legs and lacking any sort of elytral and pronotal spots as seen in *M. tigrinus*. On the other hand, *M. sallei* and *M. spadiceus* have three black pronotal spots and a band or a spot on each elytron, respectively. Although color pattern seems to be plastic in some Erotylidae lineages [25], the biological mechanism (e.g., feeding habits, polymorphism or polyphenism) underlying color determination is unknown for the family. More sampling, information on host fungi and detailed geographical distribution, combined with molecular data, are required to determine the relationships of the aforementioned species with *M. tigrinus* and make further taxonomic decisions.
